# Supplementary material for: Cell-density independent increased lymphocyte production and loss rates post-autologous HSCT
Source: eLife. 2021 Feb 4;10:e59775. doi: 10.7554/eLife.59775 (PMC7886352; doi:10.7554/eLife.59775)
Supplement: Transparent reporting form [file elife-59775-transrepform.docx]

***eLife’s* transparent reporting form**

We encourage authors to provide detailed information *within their submission* to facilitate the interpretation and replication of experiments. Authors can upload supporting documentation to indicate the use of appropriate reporting guidelines for health-related research (see [EQUATOR Network](http://www.equator-network.org/%20)), life science research (see the [BioSharing Information Resource](https://biosharing.org/)), or the [ARRIVE guidelines](http://www.plosbiology.org/article/info:doi/10.1371/journal.pbio.1000412) for reporting work involving animal research. Where applicable, authors should refer to any relevant reporting standards documents in this form.

If you have any questions, please consult our Journal Policies and/or contact us: [editorial@elifesciences.org](mailto:editorial@elifesciences.org).

**Sample-size estimation**

- You should state whether an appropriate sample size was computed when the study was being designed
- You should state the statistical method of sample size computation and any required assumptions
- If no explicit power analysis was used, you should describe how you decided what sample (replicate) size (number) to use

Please outline where this information can be found within the submission (e.g., sections or figure legends), or explain why this information doesn’t apply to your submission:

The sample size calculation was in the study proposal which was approved by the medical ethical committee of our institute, which is mentioned in the material and method section. When submitting the proposal for approval of the ethical committee we performed two different power calculations(http://www.dssresearch.com/toolkit/spcalc/power_a2.asp,2 sample t test, error level (α) 5, power 80%, same standard deviation 33% of mean of control group, based on earlier estimates in healthy controls), differences between average of means 50% increase) either two sided (calculated n=7-8) or one sided assuming that the production rate will be increased in the HSCT patients compared to healthy controls (n=5). In order to not to burden too many patients we decided to start with including 5 patients. Since the second patient who was included did not finish the protocol we decided to include one extra patient.

**Replicates**

- You should report how often each experiment was performed
- You should include a definition of biological versus technical replication
- The data obtained should be provided and sufficient information should be provided to indicate the number of independent biological and/or technical replicates
- If you encountered any outliers, you should describe how these were handled
- Criteria for exclusion/inclusion of data should be clearly stated
- High-throughput sequence data should be uploaded before submission, with a private link for reviewers provided (these are available from both GEO and ArrayExpress)

Please outline where this information can be found within the submission (e.g., sections or figure legends), or explain why this information doesn’t apply to your submission:

Trough out the paper either information of separated samples is shown, or the median of longitudinal data (biological replicates) of one participant. For the latter longitudinal data, showing all biological replicates, is shown separately as figure supplement figure or table. Control values are given as separated samples, median, 95% confidence interval or interquartile range of single samples from different healthy control. For these the N are indicated in the legends.

For the enrichment no biological replicates (within in participants) are available since the amount of blood required to prepare one analyte was too large to perform multiple draws at the same time and enrichment level differ over time. Technical replicates (performing steps within a analytical process multiple times with the same sample) where performed in the GCMS enrichment analysis and TREC analysis. In both these analyses presence of the analyte is calculated based on standard curves. Duplicates were performed and the mean of the duplicates is presented as the determined value. This is mentioned in the material and methods.

**Statistical reporting**

- Statistical analysis methods should be described and justified
- Raw data should be presented in figures whenever informative to do so (typically when N per group is less than 10)
- For each experiment, you should identify the statistical tests used, exact values of N, definitions of center, methods of multiple test correction, and dispersion and precision measures (e.g., mean, median, SD, SEM, confidence intervals; and, for the major substantive results, a measure of effect size (e.g., Pearson's r, Cohen's d)
- Report exact p-values wherever possible alongside the summary statistics and 95% confidence intervals. These should be reported for all key questions and not only when the p-value is less than 0.05.

Please outline where this information can be found within the submission (e.g., sections or figure legends), or explain why this information doesn’t apply to your submission:

Median of biological replicates or values over time are given. For comparison between two unpaired groups we assumed non Gaussian distribution and performed Mann Whitney tests. Using the same assumption we used Kruskal-Wallis with Dunn’s correction for comparison of multiple groups as mentioned in material methods and in the legends

(For large datasets, or papers with a very large number of statistical tests, you may upload a single table file with tests, Ns, etc., with reference to sections in the manuscript.)

**Group allocation**

- Indicate how samples were allocated into experimental groups (in the case of clinical studies, please specify allocation to treatment method); if randomization was used, please also state if restricted randomization was applied
- Indicate if masking was used during group allocation, data collection and/or data analysis

Please outline where this information can be found within the submission (e.g., sections or figure legends), or explain why this information doesn’t apply to your submission:

Not applicable

**Additional data files (“source data”)**

- We encourage you to upload relevant additional data files, such as numerical data that are represented as a graph in a figure, or as a summary table
- Where provided, these should be in the most useful format, and they can be uploaded as “Source data” files linked to a main figure or table
- Include model definition files including the full list of parameters used
- Include code used for data analysis (e.g., R, MatLab)
- Avoid stating that data files are “available upon request”

Please indicate the figures or tables for which source data files have been provided:

Source data files are provided for

Figure 2, 3 ,4 ,6,7 and 8
